# High seroprevalence of SARS-CoV-2 in elderly care employees in Sweden

**DOI:** 10.1080/20008686.2020.1789036

**Published:** 2020-08-05

**Authors:** Johanna F. Lindahl, Tove Hoffman, Mouna Esmaeilzadeh, Björn Olsen, Reidar Winter, Stefan Amer, Christian Molnár, Ann Svalberg, Åke Lundkvist

**Affiliations:** a Department of Medical Biochemistry and Microbiology, Zoonosis Science Center, Uppsala University, Uppsala, Sweden; b Department of Clinical Sciences, Swedish University of Agricultural Sciences, Uppsala, Sweden; c Department of Biosciences, International Livestock Research Institute, Hanoi, Vietnam; d Department of Medical Sciences, Zoonosis Science Center, Uppsala University, Uppsala, Sweden; e Department of Cardiology and Clinical Physiology, eHeart AB, Stockholm, Sweden; f Saltsjöbaden, Sweden

**Keywords:** COVID-19, SARS-CoV-2, rapid test, IgM, IgG, employees, elderly care homes

## Abstract

The COVID-19 pandemic is growing and spread in the Swedish elderly care system during April 2020. The increasing number of employees on sick-leave due to COVID-19 created severe logistic problems. Some elderly care homes therefore started to screen their personnel to secure the safety of the elderly and to avoid unnecessary quarantine of potentially immune employees.

Secondary data from a screening with a COVID-19 rapid test for detection of SARS-CoV-2-specific IgM and IgG of 1,005 employees in 22 elderly care homes in Stockholm, Sweden, were analyzed. Seropositive employees were found in 21 out of the 22 care homes. In total, 23% (231/1,005) of the employees tested positive for antibodies against SARS-CoV-2, and 14.3% (144/1,005) were found positive for IgM (either alone or combined with IgG), indicating recent or present infection. Of those that tested seropositive, 46.5% did not report any clinical symptoms, indicating pre- or asymptomatic infections. Reported symptoms with the highest correlation with seropositivity were fever and loss of smell and taste.

These results suggest that antibody testing of employees in elderly care homes is valuable for surveillance of disease development and a crucial screening tool in the effort to decrease the death toll in this pandemic.

## Background

The new viral pneumonia Coronavirus Disease 2019 (COVID-19), caused by the novel Severe Acute Respiratory Syndrome Coronavirus-2 (SARS-CoV-2), has since the first reported cases in January in Wuhan, Hubei province, China, rapidly developed into a global pandemic [1–3]. By mid-May 2020, COVID-19 has spread to all permanently inhabited continents, 213 countries, and had caused more than 4.9 million reported human cases globally, with more than 320,000 human deaths [4]. The total number of actual COVID-19 cases is likely much higher than the number of confirmed cases due to limited testing in many geographical areas.

The major clinical symptoms of COVID-19 resemble respiratory illnesses caused by other viruses, i.e. fever and dry cough [5–8]. Anosmia (loss of smell) and ageusia (loss of taste) have also been noted as early and sometimes the only symptoms [9–11]. In addition, the virus has been detected in completely asymptomatic individuals [12,13] and there are estimates that the true asymptomatic portion may be as high as 80% [14]. Increasing age is an important risk factor for fatal outcome in COVID-19, and co-morbidities such as hypertension, diabetes, and cardiovascular disease associated with poor outcomes are more common in the elderly [15–17]. The case fatality rate increases drastically after the age of 60, with >3% in patients above 60 and between 14% and 20% in those above 80 [18,19].

As in other parts of the world, COVID-19 has rapidly spread to Swedish elderly care homes and resulted in an increasing number of deaths. The Swedish government issued a formal recommendation against visiting elderly care homes on April 1 [20], in spite of the potential detrimental risks associated with a social isolation of the elderly [21]. Even so, 191 out of 313 (61.0%) elderly care homes in Stockholm had confirmed at least one case of COVID-19 among their residents by April 19 2020, and 380 out of the 921 (41.3%) COVID-19 total death toll in Stockholm occurred among the elderly care home residents [22]. In addition, the number of employees in the elderly care on sick-leave due to COVID-19 was rapidly increasing. The time point for a safe return to the workplace after COVID-19 is at present unknown, since the knowledge concerning the immunology as well as the potential increase in herd-immunity to COVID-19 is still most limited. Large serological screenings of employees at elderly care homes are therefore urgently needed, and tools are increasingly becoming available. In a recent study, we evaluated a commercially available assay, the COVID-19 IgG/IgM rapid test cassette, developed for rapid detection of SARS-CoV-2-specific antibodies and found a specificity of 100% for immunoglobulin (Ig) M and 99.2% for IgG [23]. In the present study, we analyzed secondary data from elderly care homes in Stockholm, the capital of Sweden, where employees had been screened for antibodies by this rapid test.

## Material and methods

**Figure 1.**
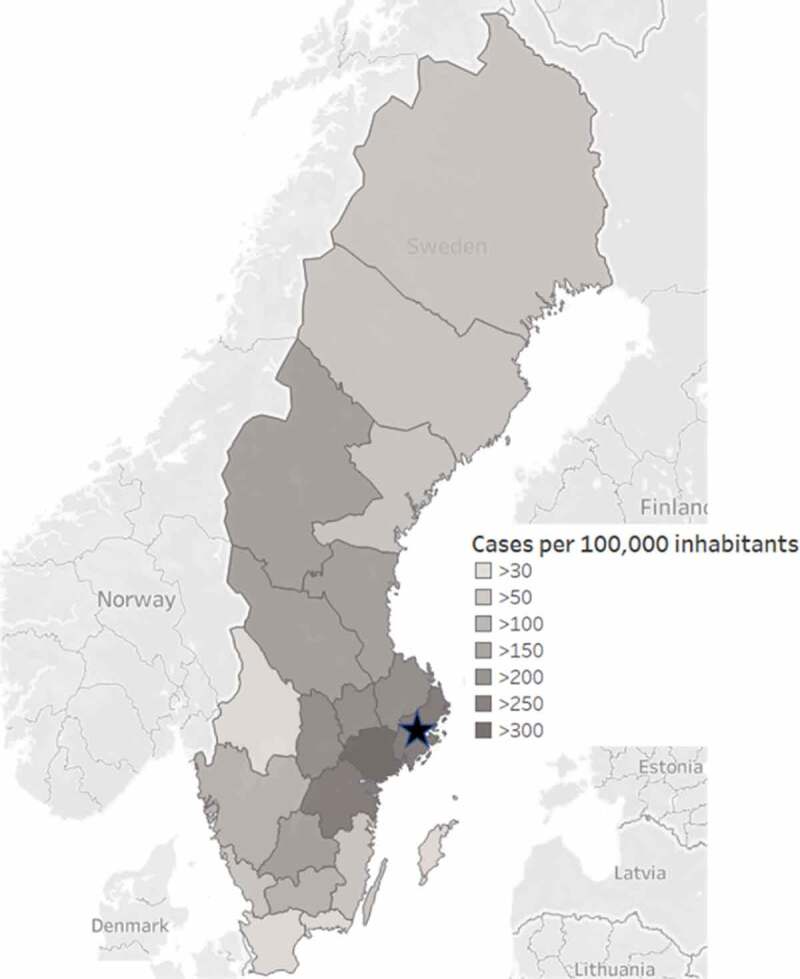
Map of the incidence of laboratory-confirmed COVID-19 cases [25] in Sweden as of April 23 2020, with the location of Stockholm marked with a star.

This study analyzed secondary data made available from elderly care homes situated in Stockholm city and its suburbs in the North, West, and South distributed between approximately 59°7ʹ24-59°25ʹ32 N and 17°45ʹ44-18°15ʹ14 E (Figure 1), where employees had been tested using the COVID-19 IgG/IgM rapid test cassette (Zhejiang Orient Gene Biotech Co Ltd, Huzhou, Zhejiang, China), which has been described previously [23,24]. This test produces a line for SARS-CoV-2-specific IgM or IgG, or both. In addition to test results, the anonymous data set included age, gender, if the employee had been in contact with someone with COVID-19, and if the employee had any COVID-19 symptoms at the time of testing or during the preceding 2 months before the sampling. Some employees also specified clinical symptoms. The elderly care homes reported different data, and for most, only test results were reported. All tests were performed during the first 20 days of April 2020.

Data were managed in Excel and STATA 14.2 (StataCorp Ltd, College Station, Texas). The variable on having any symptoms did only include observations that had data of both present and past symptoms, no imputation of missing data was made. Analyses between categorical variables were performed with logistic regression (logit command), Chi2 test, or Fisher’s exact test when the assumptions for Chi2 test were not met. T-test was used to assess associations with age. Correlation between reported symptoms was tested with command pwcorr to ensure no correlation above 0.6. A multivariable model was built for seropositivity including symptoms associated with p < 0.2 in univariable analyses, as well as contact with COVID-19 cases, using meqrlogit, with elderly care home as a random effect. The model was optimized by removing variables with higher p-value than 0.05, if they did not affect other variables with more than 25%. All data were handled anonymously, and ethical approval was given by the Swedish Ethical Review Authority (2020–02047).

## Results

The study included 1,005 employees from 22 different elderly care homes in Stockholm. Data on gender and age were available from nine of the homes, indicating that 17.0% (56/330) of the employees were males and that 40.1% (133/332) and 34.9% (116/332) belonged to the age groups 36–50 and 51–65, respectively. Data on potential contact with someone with COVID-19 were available for 331 employees from these nine homes and contacts were reported by 37.8% (125/331) and varied between the homes (p < 0.001) (Table 1). Nine employees (9/79, 11.4%) reported this contact to be private, while 70 (70/79, 88.6%) reported the contact to have been work related.

**Table 1.** Serological responses of 1,005 employees to SARS-CoV-2 at 22 different elderly care homes in Stockholm, Sweden, and the number of employees that reported known contacts with COVID-19 cases.

		Seropositive		IgM only		IgM and IgG		IgG only		Reported contact	
Home	Tested	Number	%	Number	%	Number	%	Number	%	Number	%*
1	50	12	24.0	1	2.0	7	14.0	4	8.0	24/50	48.0
2	65	9	13.8	0	0.0	6	9.2	3	4.6	22/62	35.5
3	37	8	21.6	0	0.0	5	13.5	3	8.1	14/37	37.8
4	112	32	28.6	8	7.1	13	11.6	11	9.8	20/27	74.1
5	33	8	24.2	2	6.1	6	18.2	0	0.0	6/32	18.8
6	25	10	40.0	2	8.0	5	20.0	3	12.0	0/25	0.0
7	17	5	29.4	0	0.0	4	23.5	1	5.9	10/17	58.8
8	73	15	20.5	0	0.0	10	13.7	5	6.8		
9	35	5	14.3	3	8.6	0	0.0	2	5.7		
10	7	0	0.0	0	0.0	0	0.0	0	0.0		
11	44	17	38.6	3	6.8	14	31.8	0	0.0		
12	11	2	18.2	0	0.0	2	18.2	0	0.0		
13	60	9	15.0	0	0.0	2	3.3	7	11.7		
14	51	11	21.6	1	2.0	5	9.8	5	9.8		
15	42	19	45.2	4	9.5	8	19.0	7	16.7		
16	80	15	18.8	1	1.3	7	8.8	7	8.8		
17	73	21	28.8	5	6.8	3	4.1	13	17.8		
18	22	4	18.2	1	4.5	1	4.5	2	9.1		
19	68	9	13.2	1	1.5	1	1.5	7	10.3		
20	19	2	10.5	1	5.3	0	0.0	1	5.3		
21	46	17	37.0	5	10.9	6	13.0	6	13.0	29/46	63.0
22	35	1	2.9	1	2.9	0	0.0	0	0.0	0/35	0.0
**Total**	**1005**	**231**	**23.0**	**39**	**3.9**	**105**	**10.4**	**87**	**8.7**	**125/331**	**37.8**

* Out of those answering this question

### Antibody reactivities and reported symptoms

**Figure 2.**
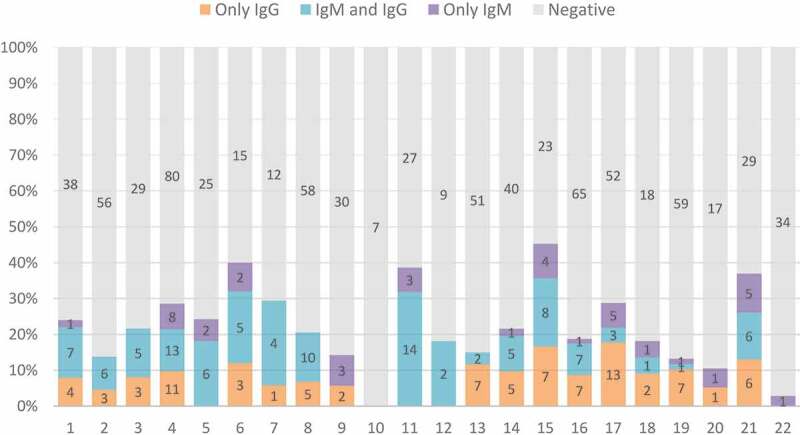
Serological results (as percentages and absolute numbers) among employees at 22 elderly care homes in Stockholm, Sweden.

Of the 1,005 employees, 231 tested positive for either IgM or IgG (23.0%; 95% confidence interval (CI): 20.4–25.7%), while 105 (10.4%; 95% CI: 8.6–12.5%) were found positive for both IgM and IgG (Table 1). Thirty-nine employees (3.9%; 95% CI: 2.8–5.3%) tested positive for only IgM while 87 (8.7%; 95% CI: 7.0–10.6%) were positive for only IgG. Out of the 231 seropositive, 45.5% were positive for both IgM and IgG, 37.7% were positive for IgG only and 16.9% were positive for only IgM. Seropositive employees were detected in all except one of the elderly care homes and the seroprevalence ranged from 0% to 45% (Figure 2, Table 1).

In total, 89.1% (409/459) of the employees, for which such data were available, reported no clinical symptom at the time of testing, while 10.9% (50/459) reported symptoms. Thirty-six percent of the employees that reported symptoms at sampling had IgM antibodies, while 24.9% of those not reporting symptoms were IgM positive (p = 0.09) (Table 2). Eighty-five percent (102/120) of the IgM positives did not have any clinical symptoms at the time of sampling, while 64.7% (33/51) had not had symptoms during the preceding month, and 45.0% (27/60) were asymptomatic 1–2 months before testing. Including observations where both present and previous symptoms were known, a total of 35.8% (123/344) of the employees had reported symptoms at some point. There were more seropositive individuals among those that had reported symptoms (37.4%, 46/123) as compared to those with no symptoms (18.1%, 40/221) (p < 0.001). However, out of the seropositive employees, 46.5% (40/86) reported having no clinical symptoms during the last two months.

**Table 2.** Prevalence of IgM (either only IgM or combined with IgG) or only IgG among elderly care home employees with or without reported symptoms.

		IgM positive (%)	IgG positive only (%)
Symptoms at sampling	Yes	18/50 (36.0)	6/50 (12.0)
	No	102/409 (24.9)	73/409 (17.8)
Symptom within the last month	Yes	18/77 (23.4)*	8/77 (10.4)
	No	33/254 (13.0)	15/254 (5.9)
Symptoms 1–2 months earlier	Yes	2/21 (9.5)	1/21 (4.8)
	No	49/310 (15.8)	22/310 (7.1)

*Significant at *p* < 0.05 compared to the row below.

In some cases, employees also reported specific symptoms (Table 3). Loss of smell and taste (anosmia and ageusia) either at time of testing or the previous month were reported by 13 employees. Out of these, 69.2% were seropositive compared to 20.4% of those not reporting these symptoms (p < 0.001). Fever was reported by 23 employees and the seroprevalence was higher among employees reporting fever as compared to those not reporting fever (p = 0.003).

**Table 3.** Predictors for seropositivity in both univariable and multivariable analyses.

			Univariable		Multivariable	
Predictor			Seropositive (%)	*p*-Value	Odds ratio (95% CI)	*p*-Value
**Contact with case**	Yes	125	40 (32.0)		2.4 (1.3–4.4)	**0.005**
	No	206	33 (16.0)			
**Reported symptoms**						
Aches	Yes	11	4 (36.4)	0.25	Not included	
	No	324	70 (21.6)			
Anosmia/ageusia	Yes	13	9 (69.2)	**<0.001**	6.4 (1.6-25.8)	**0.009**
	No	323	66 (20.4)			
Cold, sore throat	Yes	41	14 (34.2)	0.06	Not included	
	No	297	63 (21.2)			
Cough	Yes	37	12 (32.4)	0.11	Not included	
	No	298	62 (20.8)			
Dyspnea	Yes	14	6 (42.9)	0.06	Not included	
	No	321	68 (21.2)			
Fever	Yes	23	11 (47.8)	**0.003**	2.8 (1.0–8.1)	**0.049**
	No	314	65 (20.7)			
Headache	Yes	29	11 (37.9)	0.04	0.4 (0.1–1.4)	0.16
	No	309	66 (21.4)			
Tired	Yes	43	13 (30.2)	0.18	Not included	
	No	293	62 (21.2)			
					Random effect of home	0.3 (0.04–1.64)

p-Values < 0.05 of significance are indicated in bold.

### Risk factor analysis

Association with seropositivity was tested for gender, age, and contact with a COVID-19 case. Age and gender had no association with being seropositive, but those with previous contact with a COVID-19 case had an odds ratio (OR) 2.5 (95% CI: 1.5–4.2) higher than those without contact.

### Multivariable model for seropositivity

Using a mixed effects model; loss of smell, fever, and contact with COVID-19 cases were strong predictors for seropositivity (Table 3). When combined with the other factors, headache was a confounder and had a slightly negative impact on the risks.

## Discussion

In this study, we evaluated secondary data from elderly care homes in Stockholm, Sweden, where a commercial rapid test detecting SARS-CoV-2-specific IgM and IgG had been used to screen for antibody prevalence among employees. We found seropositive employees in 95.5% (21/22) of the investigated elderly care homes as well as a high seropositivity of IgM antibodies among the employees, often combined with IgG antibodies. The results indicated a recent spread of SARS-CoV-2 within elderly care in Stockholm and a potentially ongoing infection in a substantial portion of the employees. Antibody tests have lower sensitivities for recent infections [23], i.e. virus-specific antibody responses are usually not detected until a week after onset of disease, and therefore the actual seroprevalence may be slightly higher than what is estimated here. While there is a lack of publications on seroprevalence for SARS-CoV-2 in the Swedish population, there have been media reports of screenings conducted in two large hospitals, which found that 10% and 20% of employees had antibodies [26,27], indicating that elderly care employees may be an even more exposed group. A later media report of 7.3% seroprevalence in Stockholm confirms this suspicion [28].

Around half of the seropositive individuals (45% for IgM and 47% of all seropositives) in this study were pre- or asymptomatic, which is similar to the results in a study from Italy [12], but lower than the estimate of 80% [14]. Previous studies in nursing homes have found that there may be PCR-positive pre- or asymptomatic residents during an outbreak, and the same study found 19% of tested staff being positive [29]. While it is believed that most transmissions occur from symptomatic carriers through coughing or sneezing, it has also been suggested that virus could be spread through droplets or aerosols while talking [30,31], thus motivating the use of proper face masks by elderly care home employees during the pandemic. In this study, symptoms were self-reported, and data on exact symptoms were lacking for most individuals. It may be that some respondents did not want to report symptoms since they would not have been allowed to work, and therefore there may be a social desirability response bias [32], where symptoms were underreported, potentially particularly from those that tested positive. Based on the available data it seems that fever and loss of taste and smell, together with the knowledge about possible contact with a COVID-19 case, may be the best predictors for seropositivity among elderly care home employees, and it would be recommended to use molecular methods to test employees having those symptoms.

There was a correlation in the presented data between employees being seropositive and reporting contact with COVID-19 cases. Given the fragility and thus the susceptibility of the elderly to COVID-19 and the importance of securing staffing in the elderly care homes, there is a strong incitement for testing all employees in this sector. Since the rapid antibody test only detects antibodies in a qualitative manner, it would be desirable to perform sequential testing in order to monitor serological changes over time. IgM is usually a biomarker for acute infections. It might therefore seem straightforward to isolate individuals who test positive for IgM. However, our previous study using the same rapid test showed no case with IgM positivity only [23] and previous studies on SARS-CoV indicate that IgM and IgG, a biomarker for immunity, may develop almost simultaneously [33,34]. An experiment in rhesus macaques could detect IgG within 3 days post infection [35]. It would therefore be desirable to combine serological testing with a PCR test that diagnoses the virus in order to better understand the correlation between seropositivity and viral presence. However, since PCR only detects viral RNA, it would be valuable to conduct viral isolations to understand when infected individuals truly start being non-infectious. Although it is believed that IgM antibodies may develop already within some days post-infection in some individuals, the median seroconversion for IgM and IgG to SARS-CoV-2 was 12 and 14 days, respectively, in a recent study [36]. Thus, the sensitivity of any antibody tests is likely low during the first phase of the infection, which would motivate repeated testing. A SARS-CoV-2 infection is likely followed by immunity [35], and developed antibodies have been demonstrated to be neutralizing (inhibiting viral replication) using plaque reduction neutralization test (PRNT) and microneutralization tests [37–39] but the duration and extent of immunity is at present unknown and should be investigated during the progression of the pandemic, as well as the role of different antibody classes, including IgA.

Given the urgency of the situation in the health and elderly care, the present study proved antibody testing to be a fast and efficient way to map and isolate infected employees in order to prevent transmission, as well as to secure staffing by revealing those with IgG antibodies and potential immunity. However, more research on the development of antibodies during COVID-19, combined with molecular studies, is needed in order to make proper recommendations on when exposed staff can safely return to work. With the elderly of 80 years of age and older having a case fatality rate of potentially as high as 20% [19], this risk group needs to be protected as much as possible.

In conclusion, our results indicate a high prevalence of antibodies against SARS-CoV-2 and a high percentage of pre- or asymptomatic infections among employees in elderly care homes, and thus a risk for the elderly to become infected by their care givers. Employees must therefore be provided with proper personal protective equipment, and training in how to used it in order to prevent further spread of the virus in the elderly care settings.
